# Investigating radical cation chain processes in the electrocatalytic Diels–Alder reaction

**DOI:** 10.3762/bjoc.14.51

**Published:** 2018-03-16

**Authors:** Yasushi Imada, Yohei Okada, Kazuhiro Chiba

**Affiliations:** 1 Department of Applied Biological Science, Tokyo University of Agriculture and Technology, 3-5-8 Saiwai-cho, Fuchu, Tokyo 183-8509, Japan; 2 Department of Chemical Engineering, Tokyo University of Agriculture and Technology, 2-24-16 Naka-cho, Koganei, Tokyo 184-8588, Japan

**Keywords:** chain process, Diels–Alder reaction, electrocatalytic, radical cation, single electron transfer

## Abstract

Single electron transfer (SET)-triggered radical ion-based reactions have proven to be powerful options in synthetic organic chemistry. Although unique chain processes have been proposed in various photo- and electrochemical radical ion-based transformations, the turnover number, also referred to as catalytic efficiency, remains unclear in most cases. Herein, we disclose our investigations of radical cation chain processes in the electrocatalytic Diels–Alder reaction, leading to a scalable synthesis. A gram-scale synthesis was achieved with high current efficiency of up to 8000%. The reaction monitoring profiles showed sigmoidal curves with induction periods, suggesting the involvement of intermediate(s) in the rate determining step.

## Introduction

Recently, radical ion reactivity has received great attention in the field of synthetic organic chemistry. The single electron transfer (SET) strategy is the key to generating radical ions, which provide powerful intermediates for bond formations. Photo- [1–6] and electrochemistry [7–12] are the most straightforward approaches to induce SET processes. Since the pioneering work by Ledwith [13–17], a chain process involving radical ions has constituted a unique mechanism for this class of reactions, which also has the potential for contributing to effective catalytic transformations. Although understanding the chain mechanism is a prerequisite to the rational design of new radical ion-based reactions, it remains unclear in most cases. In particular, only a handful of reports have mentioned the “length,” also referred to as catalytic efficiency, of such radical ion chain processes. As an early example, Bauld estimated the chain lengths of cyclodimerizations of cyclohexadiene and *trans*-anethole (**1**) [18]. More recently, Yoon has established a straightforward method to estimate the chain length of photoredox processes using the combination of quantum yield and luminescence quenching experiments [19]. With such an understanding in hand, radical ion chain processes could be further optimized to realize greener transformations.

We have been developing anodic cycloadditions [20–25] enabled by lithium perchlorate/nitromethane electrolyte solution [26], some of which were achieved with a catalytic amount of electricity. Such electrocatalytic cycloadditions should involve radical cation chain processes, meaning that the reaction is not only triggered by an oxidative SET at the surface of the electrode but also by an intermolecular SET process in bulk solution. We assumed that the catalytic efficiency of the reaction would be further improved through optimizing and/or tuning the conditions in order to facilitate the bulk SET processes. As recently demonstrated by Baran [27–28] and Waldvogel [29–30], electrochemical synthesis has also proven to be highly scalable as well as sustainable. The longer chain length, also referred to as a higher “current efficiency” in this context, would enhance such advantages of the electrochemical synthesis. It should also be noted that the mechanism of electrochemical reactions can easily be studied since the amount of electricity passed can be precisely controlled in a switchable manner. Described herein is a demonstration of excellent current efficiency and high productivity for the electrocatalytic Diels–Alder reaction.

## Results and Discussion

**Scheme 1:**
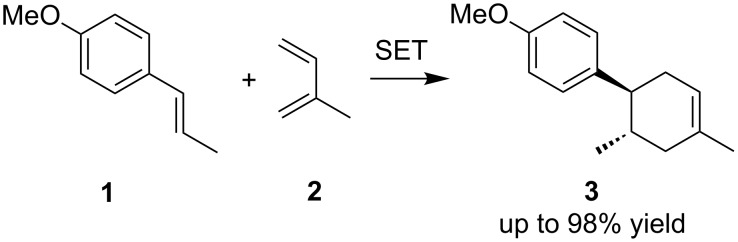
SET-triggered Diels–Alder reaction of *trans*-anethole (**1**) and isoprene (**2**).

**Figure 1:**
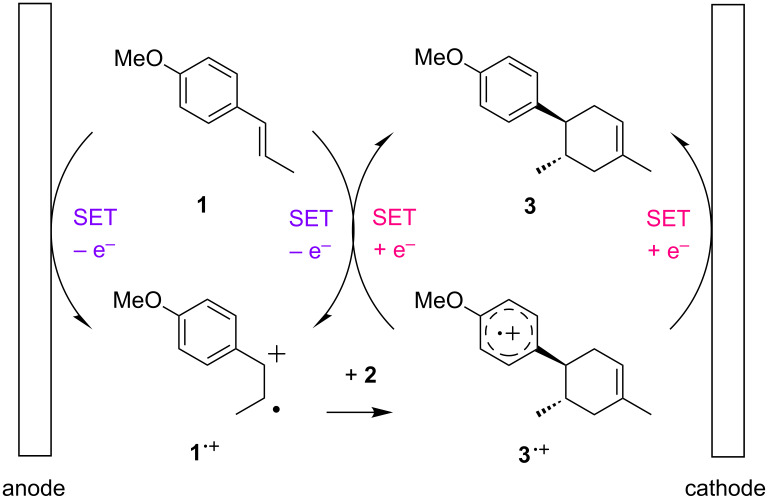
Plausible mechanism for the electrocatalytic Diels–Alder reaction. Adapted from [22].

The present work began by optimizing the SET-triggered Diels–Alder reaction of *trans*-anethole (**1**) and isoprene (**2**) as models (Scheme 1), which was first reported by Bauld [31] and was elegantly revisited by Yoon [32]. We also reported the electrochemical variation of the reaction [21–22], which was found to go to completion with a catalytic amount of electricity. The desired Diels–Alder adduct **3** was obtained in 98% yield with less than 0.1 F/mol of electricity, suggesting that the current efficiency was up to 980%. The key step in the chain process should be the bulk SET between the starting *trans*-anethole (**1**) and the aromatic radical cation (**3^·+^**), triggering the catalytic cycle (Figure 1). The concentration of substrates must be balanced to lengthen the chain process, since a higher concentration of *trans*-anethole (**1**) is effective for the bulk SET, but it can also cause undesired self-dimerization. We intentionally stopped the reaction at 0.01 F/mol in order to highlight the difference between the concentrations used and then optimize them (Table 1).

**Table 1:** Optimization of the electrocatalytic Diels–Alder reaction.

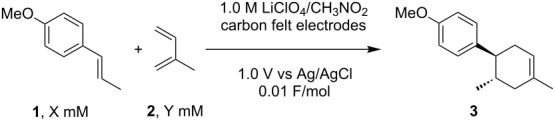		

Entry^a^	Conditions	Yield (%)^b^, current efficiency (%)

1	X = 16, Y = 48	13, 1300
2	X = 100, Y = 300	58, 5800
3	X = 300, Y = 900	62, 6200
4	X = 300, Y = 3000	80, 8000

^a^All reactions were carried out in 10 mL of CH_3_NO_2_ at rt. ^b^Yields were determined by ^1^H NMR analysis using benzaldehyde as an internal standard.

**Scheme 2:**
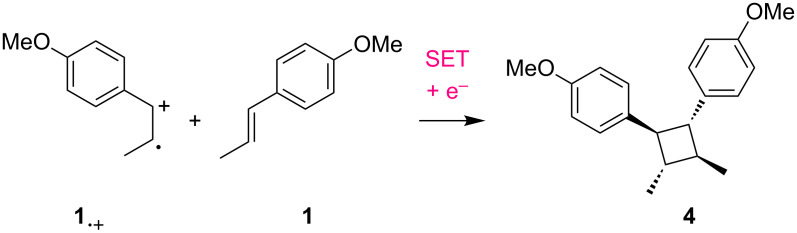
Undesired dimerization of *trans*-anethole (**1**).

When a relatively lower concentration was used, the current efficiency was measured at 1300% (Table 1, entry 1). The termination of the radical cation chain process would be a reductive SET of the radical cations (**1^·+^**, **3^·+^**) at the cathode. Therefore, we tested higher concentrations of the substrates to facilitate the bulk SET processes. Gratifyingly, we were able to achieve increased current efficiencies when 100 mM and 300 mM concentrations of *trans*-anethole (**1**) were used (Table 1, entries 2 and 3). However, some dimerization of *trans*-anethole (**1**) via the trapping of the radical cation (**1^·+^**) by neutral *trans*-anethole was also detected in both cases (Scheme 2). Since the undesired dimerization can decrease the current efficiency, we raised the amount of isoprene (**2**) from 3 to 10 equivalents in order to suppress it (Table 1, entry 4). As a result, the current efficiency reached up to 8000%, meaning that one electron was able to produce 80 molecules of the Diels–Alder adduct **3**.

**Figure 2:**
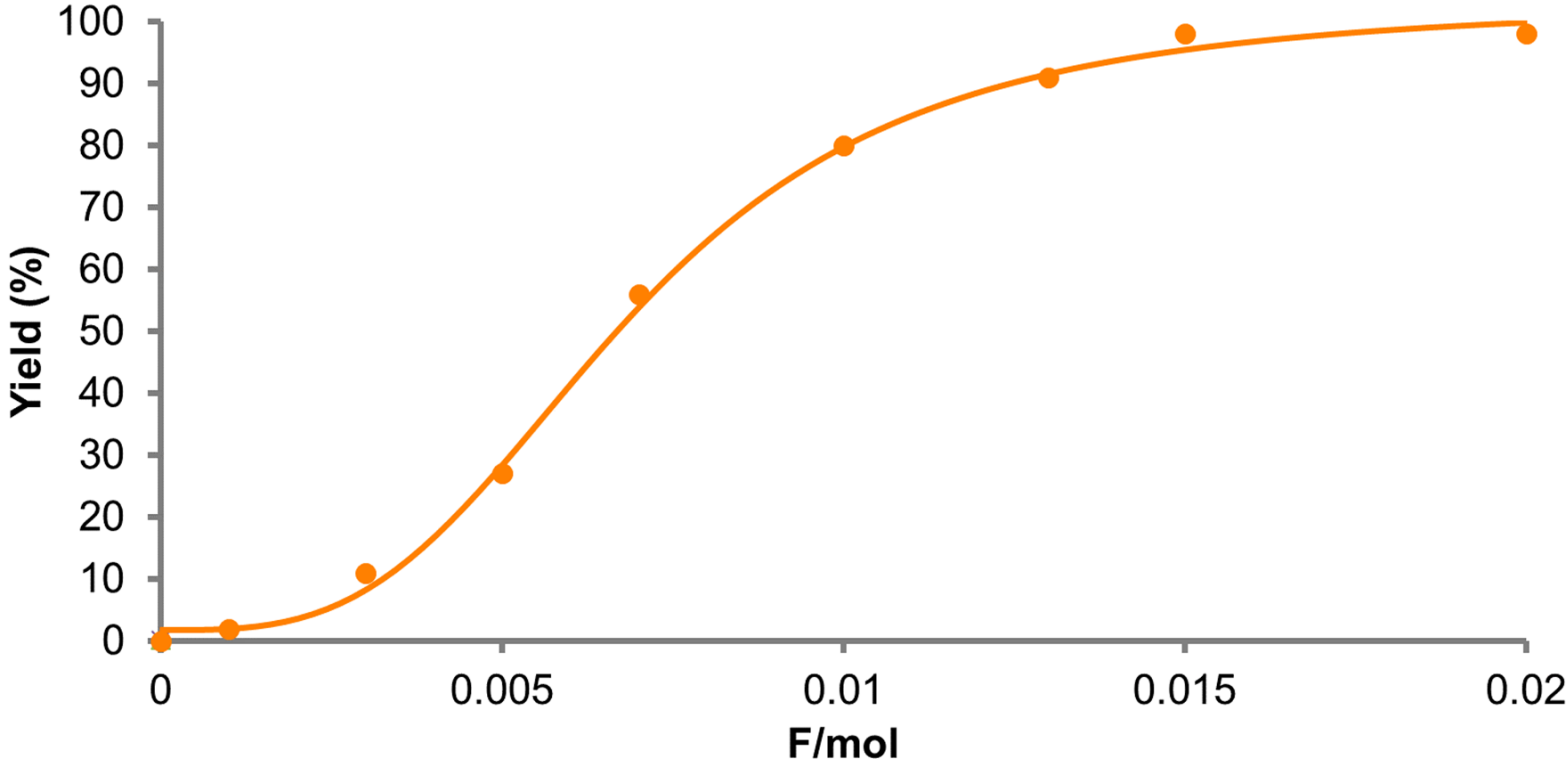
GC–MS monitoring of the electrocatalytic Diels–Alder reaction (**1**: 300 mM; **2**: 3000 mM).

With the optimized conditions (Table 1, entry 4) in hand, we monitored the reaction by gas chromatography mass spectrometry (GC–MS, Figure 2). Surprisingly, we found that only 0.015 F/mol was sufficient to complete the reaction and the Diels–Alder adduct **3** was obtained in 98% yield, suggesting that the current efficiency was up to 6533%. To the best of our knowledge, this is one of the highest current efficiencies in electrochemical synthesis in that one electron can run the radical cation chain process up to 65 times.

**Figure 3:**
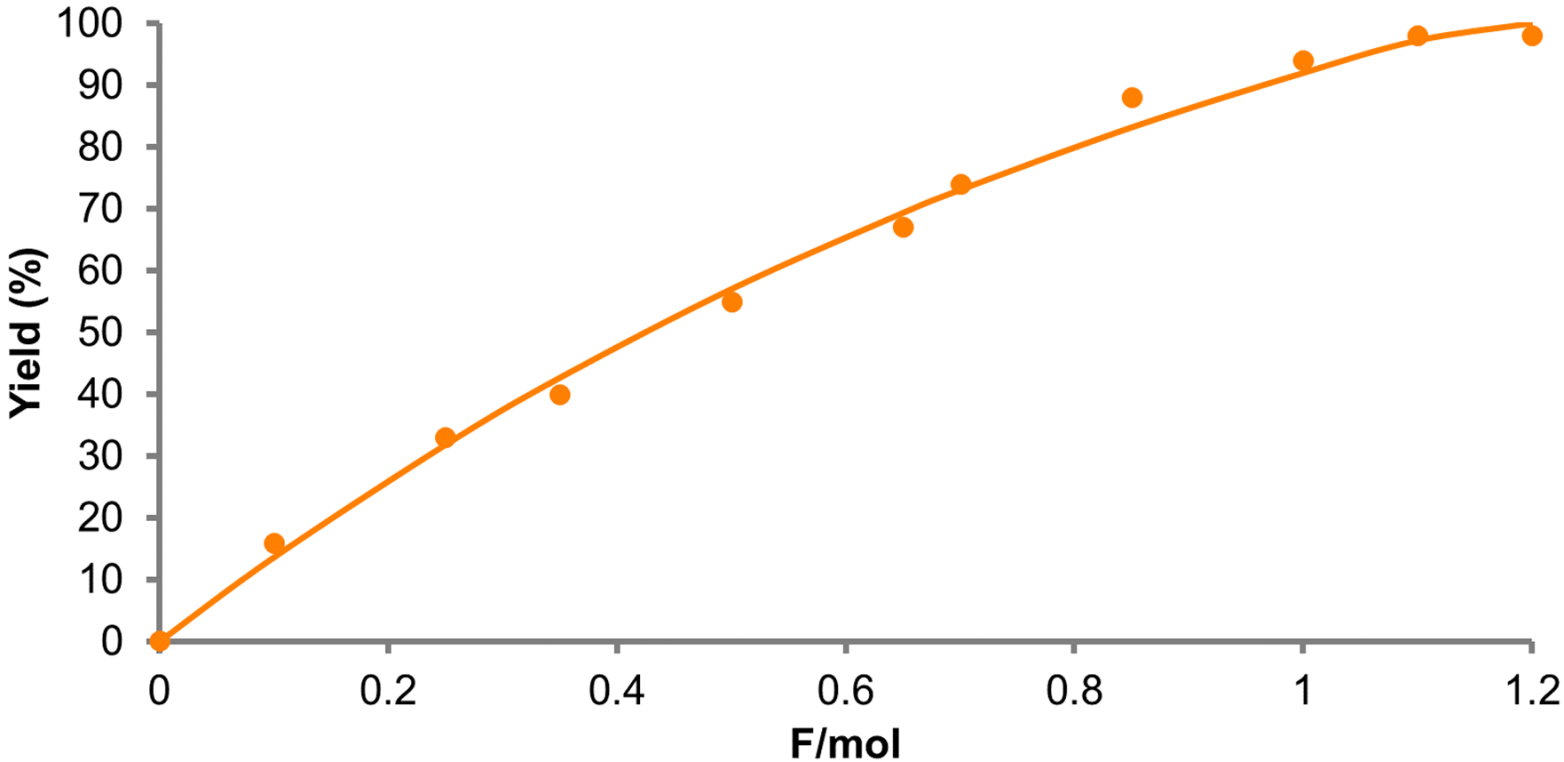
GC–MS monitoring of the electrocatalytic Diels–Alder reaction (**1**: 1 mM; **2**: 2000 mM).

GC–MS monitoring showed a sigmoidal curve with an induction period. The monitoring was carried out in the presence of a large excess (10 equivalents) of isoprene (**2**), which might follow pseudo-first order reaction kinetics. However, the observed induction period indicated that this was not the case and an intermediate(s) was involved in the rate determining step. This mechanism could be rationalized on the basis of a radical cation chain process since the concentrations of both starting *trans*-anethole (**1**) and the aromatic radical cation (**3^·+^**) would impact the overall reaction rate. Indeed, when the monitoring was carried out with a lower concentration of *trans*-anethole (**1**), no induction period was observed and the reaction required nearly a stoichiometric amount of electricity to complete (Figure 3).

**Figure 4:**
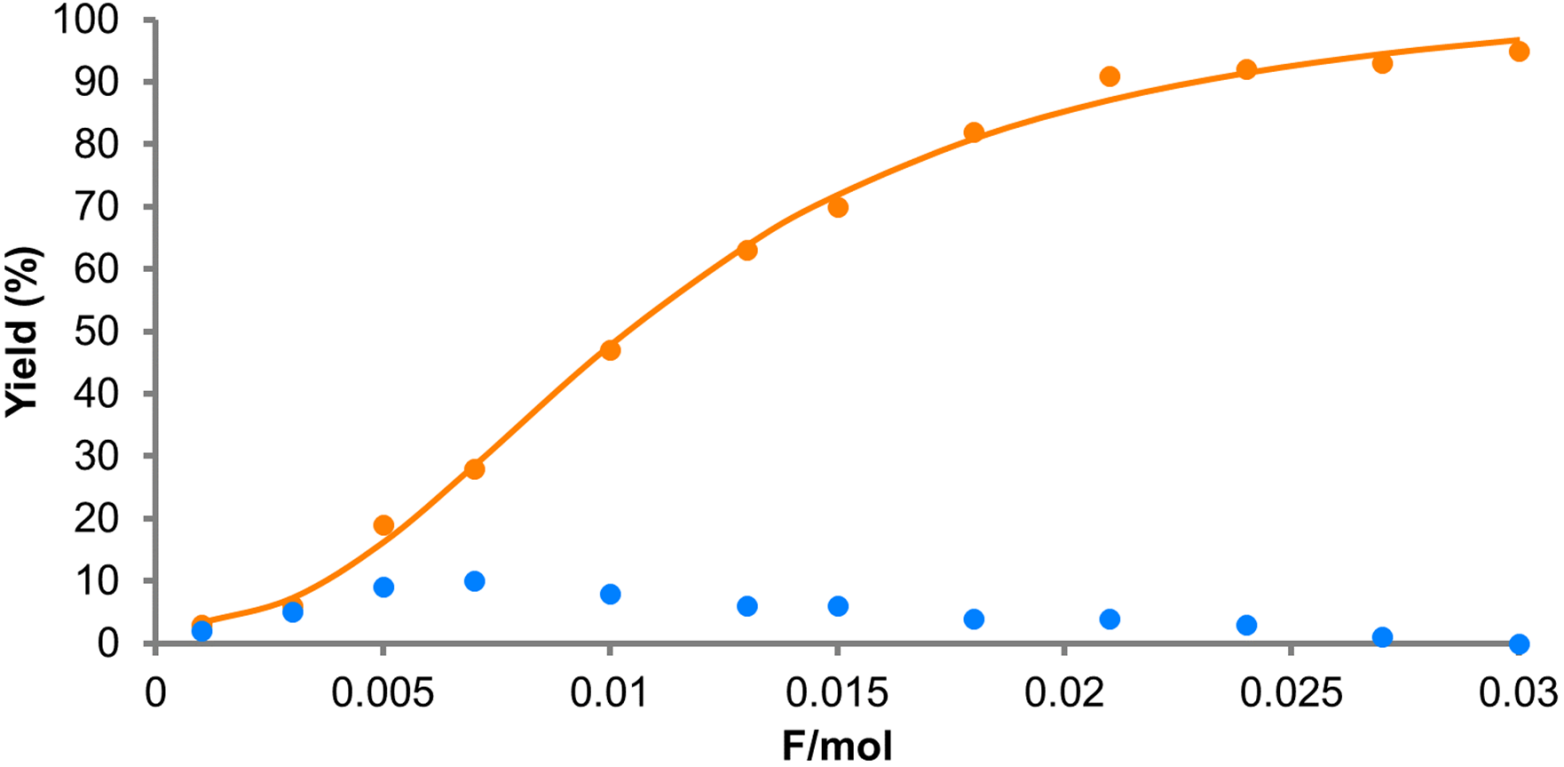
GC–MS monitoring of the electrocatalytic Diels–Alder reaction (**1**: 2 M; **2**: 6 M). Blue dots show the dimer **4**.

We finally turned our attention to the scalability of the reaction. On the basis of the above discussed radical cation chain mechanism, even higher concentrations of the substrates would still be effective (Table 2). To our delight, a high current efficiency of around 5000% was maintained even at 1.0 M and 2.0 M concentrations of *trans*-anethole (**1**, Table 2, entries 1 and 2). However, 5.0 M was too concentrated and caused precipitation of the supporting electrolyte (Table 2, entry 3). To our satisfaction, up to 4.15 g of the Diels–Alder adduct (**3**) was isolated with 0.03 F/mol, giving a current efficiency of 3167% (Table 2, entry 4). Under these conditions, the undesired dimerization of *trans*-anethole (**1**) took place to give the dimer **4**. However, we found that the electrochemical radical cation Diels–Alder reaction was also possible from the dimer, whose photochemical version was reported by Yoon [33]. The reaction was monitored by GC–MS and also showed a sigmoidal curve (Figure 4).

**Table 2:** Optimization of the electrocatalytic Diels–Alder reaction.

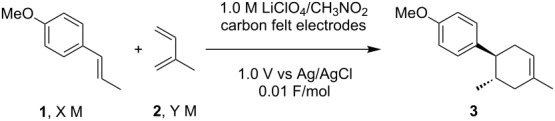		

Entry^a^	Conditions	Yield (%)^b^, current efficiency (%)

1	X = 1, Y = 3	55, 5500
2	X = 2, Y = 6	49, 4900
3	X = 5, Y = 15	2, 200
4	X = 2, Y = 6	95, 3167^c^, 4.15 g

^a^All reactions were carried out in 10 mL of CH_3_NO_2_ at rt. ^b^Yields were determined by ^1^H NMR analysis using benzaldehyde as an internal standard. ^c^0.03 F/mol was used.

## Conclusion

In conclusion, we have optimized the reaction conditions for the electrocatalytic Diels–Alder reaction in order to improve the turnover number of the radical cation chain process, which resulted in a current efficiency of up to 8000%. To the best of our knowledge, this is one of the highest current efficiencies in electrochemical synthesis to date in that one electron can run the radical cation chain process up to 80 times. Taking advantage of this effective radical cation chain process, we also demonstrated that over 4 grams of the Diels–Alder adduct were obtained with high current efficiency. The reaction was monitored by GC–MS to show sigmoidal curves, suggesting the involvement of intermediate(s) in the rate determining step. Our findings described herein would lead to further design of SET-triggered radical ion-based reactions involving chain processes with high catalytic efficiency and productivity.

## Supporting Information

File 1 Additional figure, general remarks, synthesis and characterization data, including copies of ^1^H and ^13^C NMR spectra.
