# Supplementary material for: Investigating radical cation chain processes in the electrocatalytic Diels–Alder reaction
Source: Beilstein J Org Chem. 2018 Mar 16;14:642–7. doi: 10.3762/bjoc.14.51 (PMC5870144; doi:10.3762/bjoc.14.51)
Supplement: File 1 — Additional figure, general remarks, synthesis and characterization data, including copies of 1H and 13C NMR spectra. [file Beilstein_J_Org_Chem-14-642-s001.pdf]

Supporting Information

for

Investigating radical cation chain processes in the electrocatalytic Diels–Alder reaction

Yasushi Imada[1], Yohei Okada[2], and Kazuhiro Chiba*[1]

Address: [1]Department of Applied Biological Science, Tokyo University of Agriculture and Technology, 3-5-8 Saiwai-cho, Fuchu, Tokyo 183-8509, Japan and [2]Department of Chemical Engineering, Tokyo University of Agriculture and Technology, 2-24-16 Naka-cho, Koganei, Tokyo 184-8588, Japan

Email: Kazuhiro Chiba - chiba@cc.tuat.ac.jp

Tel: (+81)-42-367-5667

Fax: (+81)-42-367-5667

*Corresponding author

Additional figure, general remarks, synthesis and characterization data, including copies of ^{1}H and ^{13}C NMR spectra

Table of contents

1. Additional figure p. S2

2. General remarks p. S3

3. Synthesis and characterization data p. S4

4. Copies of ^{1}H and ^{13}C NMR spectra p. S6

1. Additional figure

$$ce[\%] = \frac{Qtheor.}{Qpract.} \bullet \frac{nprodct}{nsubstrate} \bullet 100$$

Q = passed charge, n = amount of substrate

Figure S1. Definition of current efficiency.

2. General remarks

All reagents and solvents were purchased from commercial sources and used without further purification. Reactions were monitored by thin-layer chromatography (TLC) carried out on silica gel plates, with detection by UV absorption (254 nm) and by heating the plates after dipping them in a solution of 12 molybdo(VI)phosphoric acid *n*-hydrate in 95% ethanol. Silica gel (particle size 40−50 μm) was used for column chromatography. ^{1}H NMR spectra were collected on 600 or 400 MHz NMR spectrometers using the deuterated solvent as an internal deuterium reference. Chemical shift data are given in δ units calibrated with residual protic solvent. The multiplicity of a signal is indicated as follows: s, singlet; d, doublet; t, triplet; q, quartet; quin, quintet; m, multiplet. ^{13}C NMR spectra were collected on 150 or 100 MHz spectrometers with proton decoupling using the deuterated solvent as an internal carbon reference. Chemical shift data are given in δ units calibrated with residual solvent.

3. Synthesis and characterization data

General procedure for bulk electrolysis

*4'-Methoxy-2,4-dimethyl-1,2,3,6-tetrahydro-1,1'-biphenyl (**3**)*

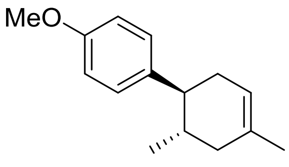

3

To a solution of lithium perchlorate (1.06 g, 10.0 mmol) in nitromethane (10 mL) stirred at rt was added *trans*-anethole (**1**) (150 μL, 1.0 mmol) and isoprene (**2**) (303 μL, 3.0 mmol). The resulting reaction mixture was electrolyzed at rt using carbon felt electrodes (2 cm × 2 cm) in an undivided cell with stirring at a constant potential of 1.0 V vs Ag/AgCl. After completion (monitored by TLC and GC–MS), the reaction mixture was diluted with CH_2Cl_2 and washed with brine. The organic layer was dried over $MgSO_4$, filtered, and concentrated in vacuo to give a crude product as a yellow oil. Silica gel column chromatography (EtOAc/Hex = 1:30) gave the title compound (211.6 mg, 98%). ^{1}H NMR ($CDCl_3$, 600 MHz) δ 7.08 (2H, d, *J* = 8.5 Hz), 6.84 (2H, d, *J* = 8.5 Hz), 5.44 (1H, br. s), 3.79 (3H, s), 2.32-2.27 (1H, m), 2.24-2.10 (2H, m), 2.06 (1H, dd, *J* = 17.2, 4.5 Hz), 1.93-1.85 (1H, m), 1.83-1.76 (1H, m), 1.69 (3H, s), 0.70 (3H, d, *J* = 6.8 Hz); ^{13}C NMR (150 MHz, $CDCl_3$) δ 157.7, 138.2, 133.8, 128.5, 120.9, 113.7, 55.2, 46.9, 39.8, 35.3, 34.0, 23.4, 20.3.

*4,4'-(3,4-Dimethylcyclobutane-1,2-diyl)bis(methoxybenzene) (**4**)*

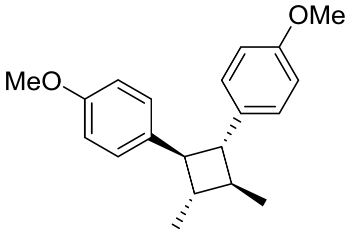

4

To a solution of lithium perchlorate (1.06 g, 10.0 mmol) in nitromethane (10 mL) stirred at rt was added *trans*-anethole (**1**) (150 μL, 1.0 mmol). The resulting reaction mixture was electrolyzed at rt using carbon felt electrodes (2 cm × 2 cm) in an undivided cell with stirring at a constant potential of 1.0 V vs Ag/AgCl. After the passage of 0.4 F/mol, the reaction mixture was diluted with CH_2Cl_2 and washed with brine. The organic layer was dried over $MgSO_4$, filtered, and concentrated in vacuo to give crude product as a yellow oil. Silica gel column chromatography (EtOAc/Hex = 1:30) gave the title compound (103.5 mg, 35%). Analytical data are in agreement with the literature.[1]

(1) Riener, M.; Nicewicz, D. A. *Chem. Sci*. **2013**, 4, 2625−2629.

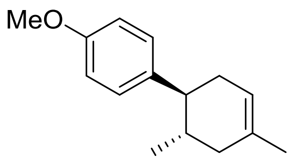
MeO

3

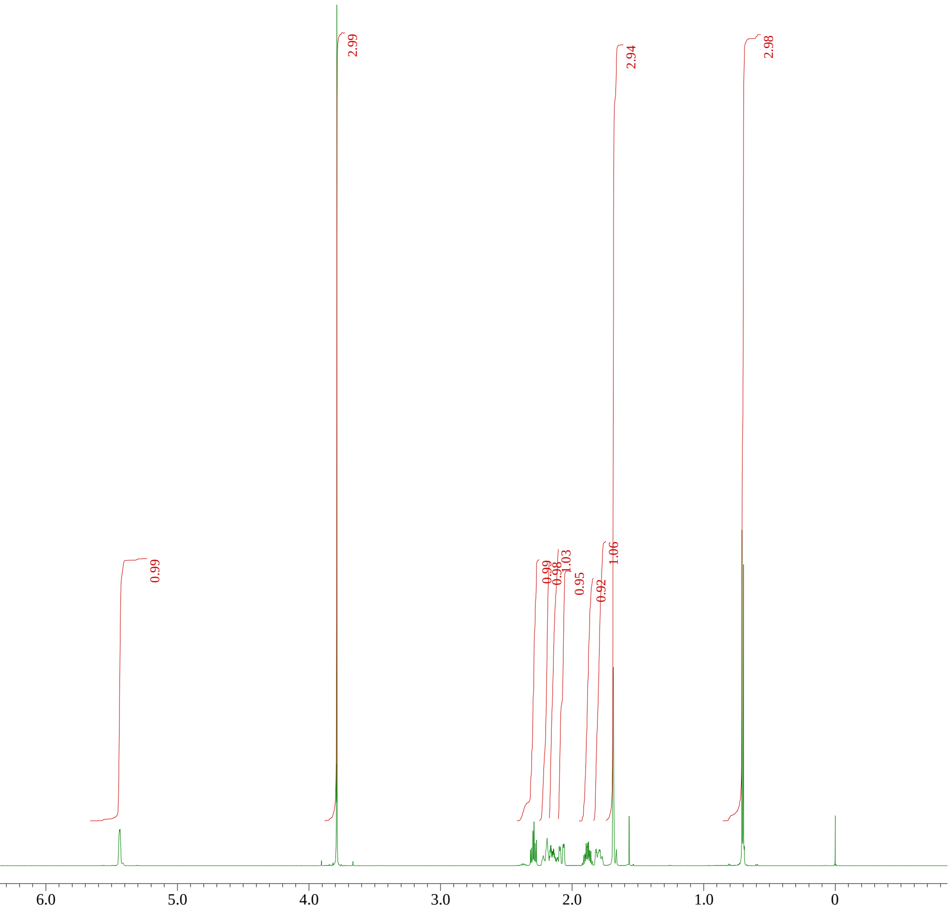
2.00
1.98
0.99
2.99
0.99
0.98
1.03
0.95
0.92
1.06
2.94
2.98
8.0
7.0
6.0
5.0
4.0
3.0
2.0
1.0
0

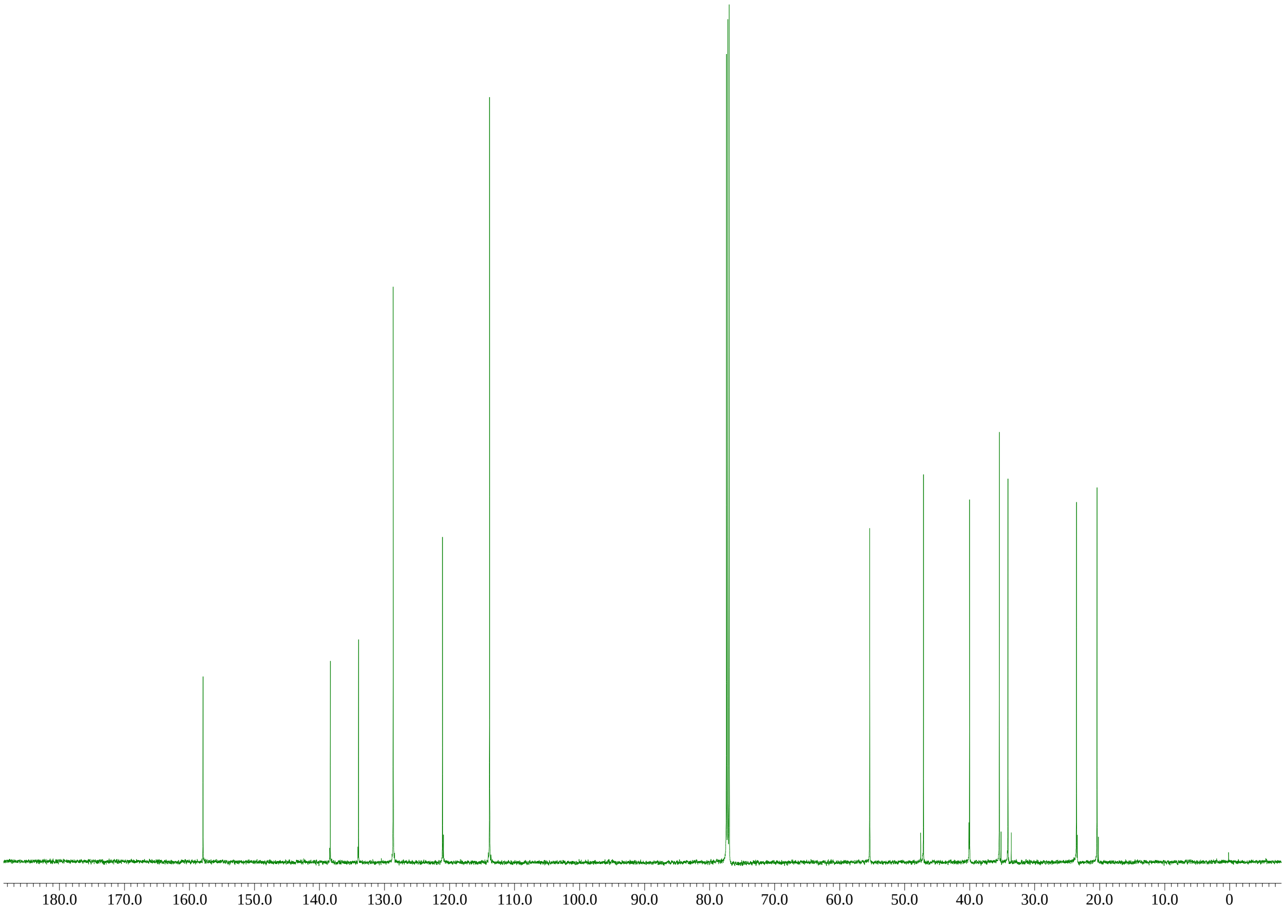
180.0
170.0
160.0
150.0
140.0
130.0
120.0
110.0
100.0
90.0
80.0
70.0
60.0
50.0
40.0
30.0
20.0
10.0
0

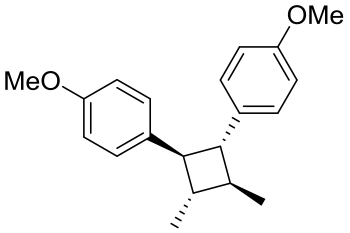

OMe
MeO

4

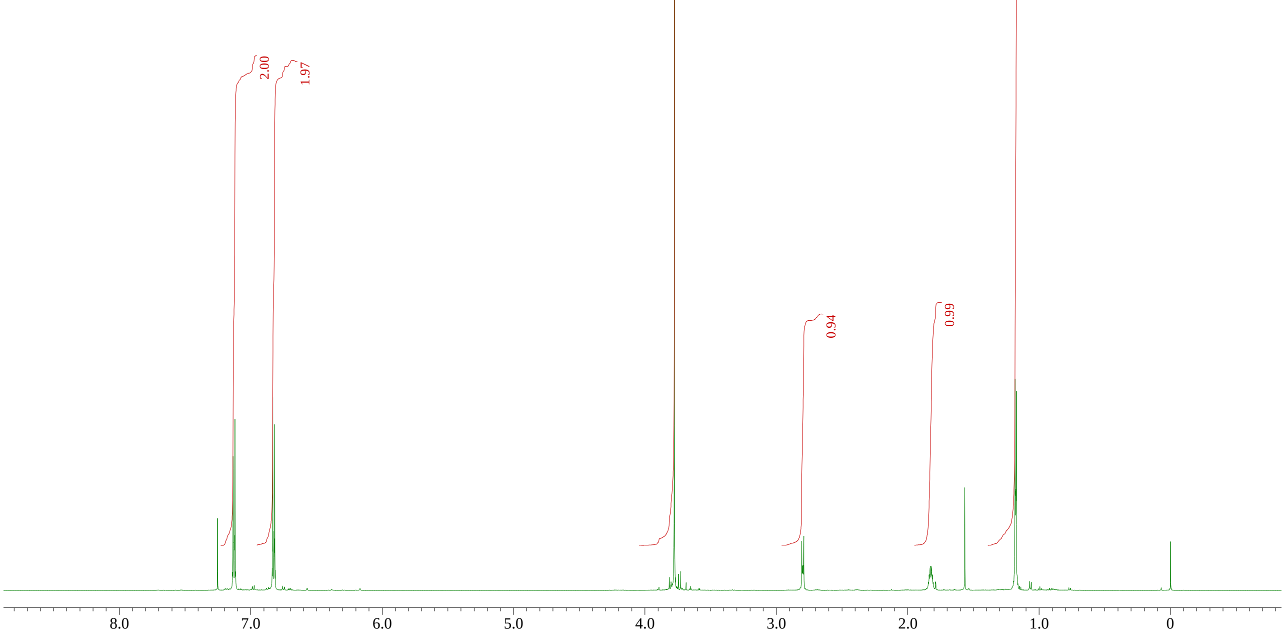

3.21
2.91
2.00
1.97
0.94
0.99
8.0
7.0
6.0
5.0
4.0
3.0
2.0
1.0
0

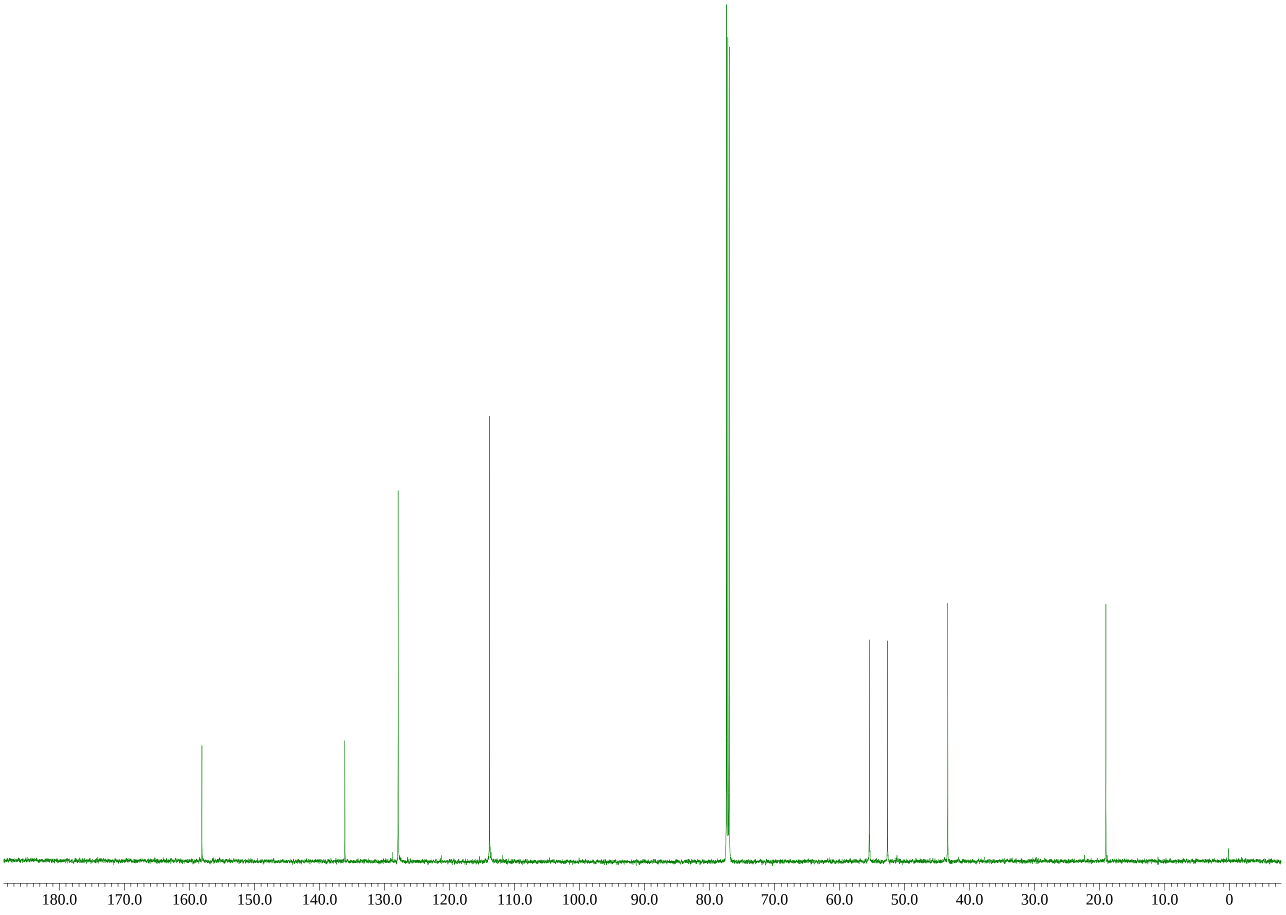

180.0
170.0
160.0
150.0
140.0
130.0
120.0
110.0
100.0
90.0
80.0
70.0
60.0
50.0
40.0
30.0
20.0
10.0
0
